# Supplementary material for: High-quality mouse reference genomes reveal the structural complexity of the murine protein-coding landscape
Source: Cell Genom. 2025 Dec 1;6(2):101074. doi: 10.1016/j.xgen.2025.101074 (PMC12903361; doi:10.1016/j.xgen.2025.101074)
Supplement: Document S1. Figures S1–S9 [file mmc1.pdf]

Cell Genomics, Volume *6*

Supplemental information

High-quality mouse reference genomes reveal the structural complexity of the murine protein-coding landscape

Mohab Helmy, Jin U. Li, Xinyu F. Yan, Rachel K. Meade, Elizabeth Anderson, Patrick B. Chen, Anne M. Czechanski, Tomás Di Domenico, Jonathan Flint, Erik Garrison, Marco T. P. Gontijo, Andrea Guarracino, Leanne Haggerty, Edith Heard, Kerstin Howe, Narendra Meena, Fergal J. Martin, Eric A. Miska, Isabell Rall, Navin B. Ramakrishna, Alexandra Sapetschnig, Swati Sinha, Diandian Sun, Francesca F. Tricomi, Runjia Qu, Jonathan M. D. Wood, Tianzhen Wu, Dian J. Zhou, Laura Reinholdt, David J. Adams, Clare M. Smith, Jingtao Lilue, and Thomas M. Keane

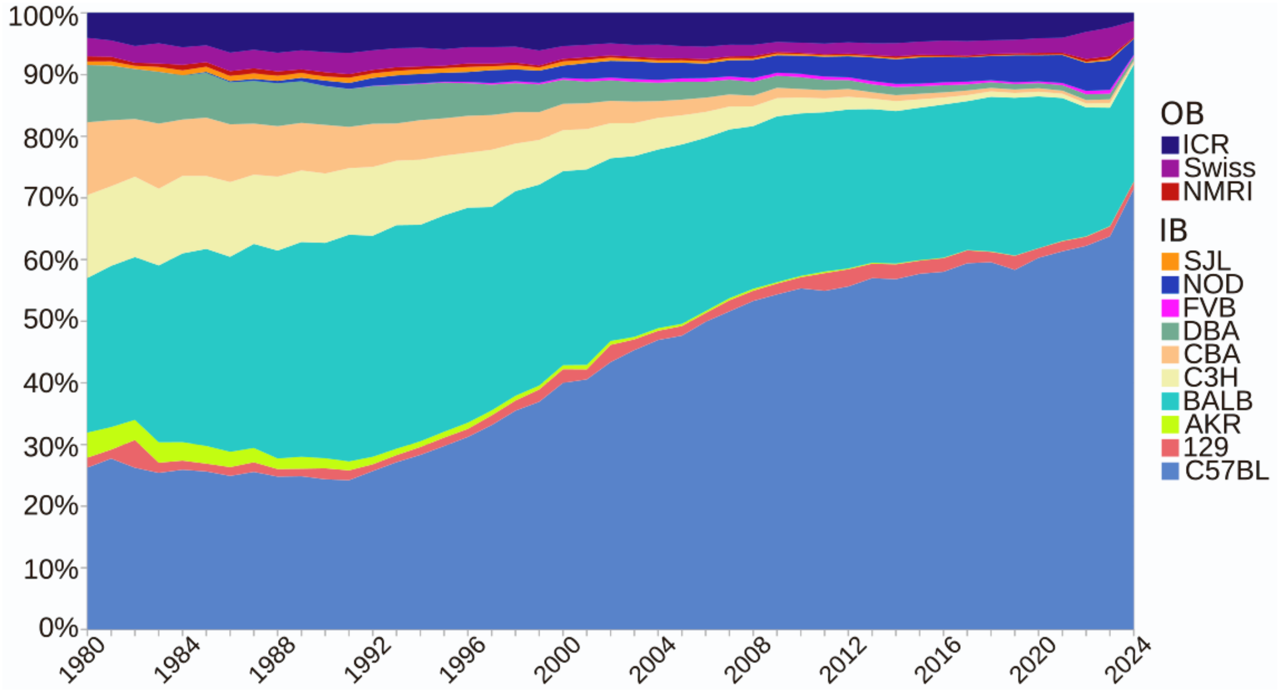

Supplementary Figure 1: PubMed publication records among the 13 widely used inbred (IB) and outbred (OB) mouse strains. The selection of publications is based on keywords in the titles and abstracts. This has led to the rediscovery of non-reference (C57BL) genes in Figure 2c.

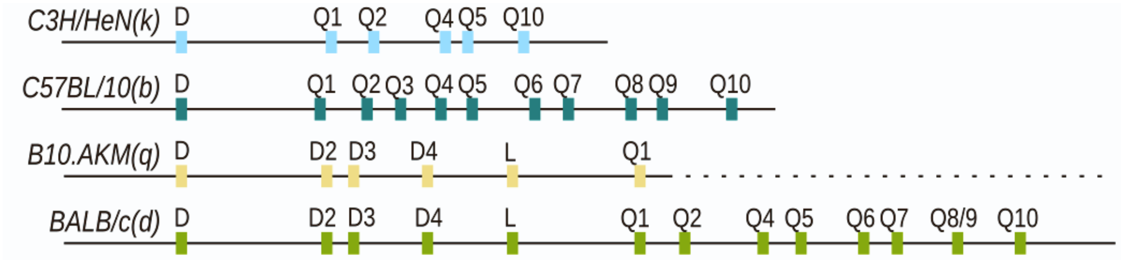

Supplementary Figure 2: Structure of H2-D/L/Q locus from haplotype *b*,*d*,*k*, and *q*, from plasmid and fosmid results. Haplotype *q* only has a partially resolved structure. The colour code is identical to Figure 2 a/b. Data are based and modified from previous publications, cited in the main text.

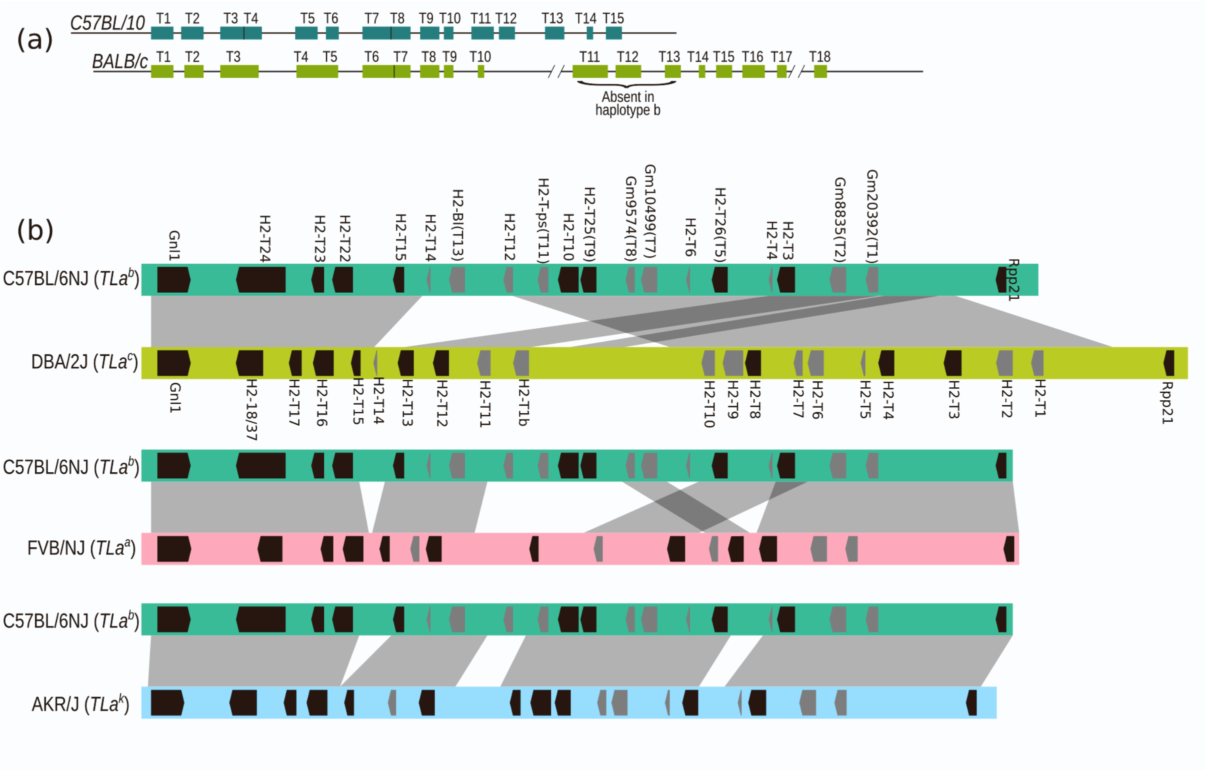

Supplementary Figure 3: Genome structure of H2-T locus in haplotype *a,b,c* and *k*. (a) genome structure from plasmid / fosmid-based data. TLa^{c} (BALB/cJ and DBA/2J) has additional copies of H2-T. Note that there is a shift in H2-T allele nomenclature between TLa^{b} and TLa^{c}. Data are based and modified from previous publications, cited in the main text. (b) Synteny of TLa^{a} (FVB/NJ), TLa^{c} (DBA/2J) and TLa^{k}(AKR/J) to TLa^{b}. The colour code is identical to Figure 2 a/b. The gene nomenclatures of TLa^{a} and TLa^{c} are based on (a).

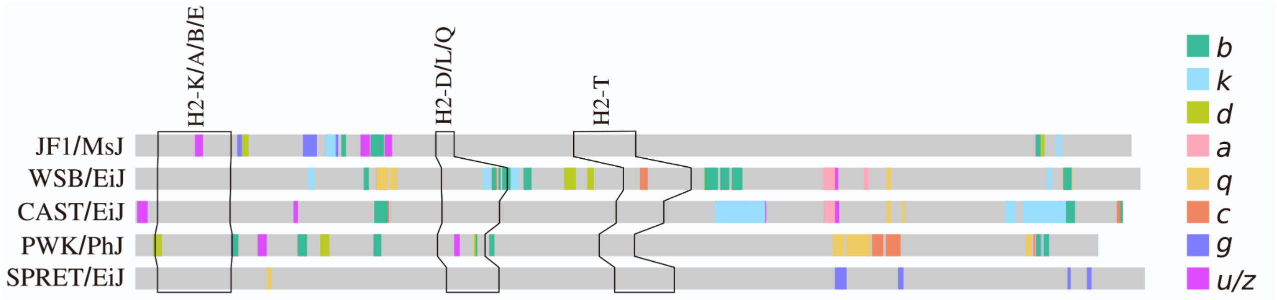

Supplementary Figure 4: Similarity between five wild-derived strains and eight known H2 haplotypes in classical laboratory mouse strains. The same haplotype is defined by 3 SNPs in 10 Kb sliding windows. The colour codes are the same as Figure 2a, and grey indicates new haplotypes in wild-derived strains. Notably the same grey colour does not mean the same haplotype.

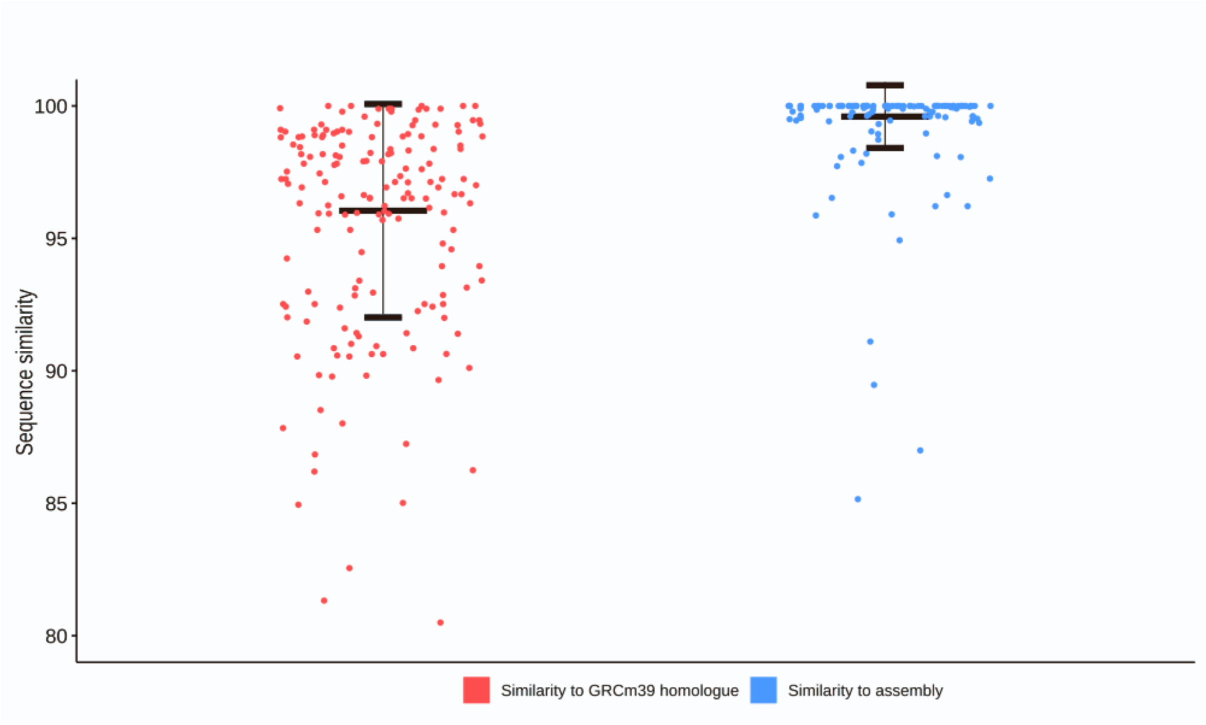

Supplementary Figure 5: Sequence similarity of 196 non-reference genes from publications to their homologues on GRCm39 reference genome (red) and novel sequence on the strain-specific *de novo* assemblies (blue), similar to those identified in Figure 2c.

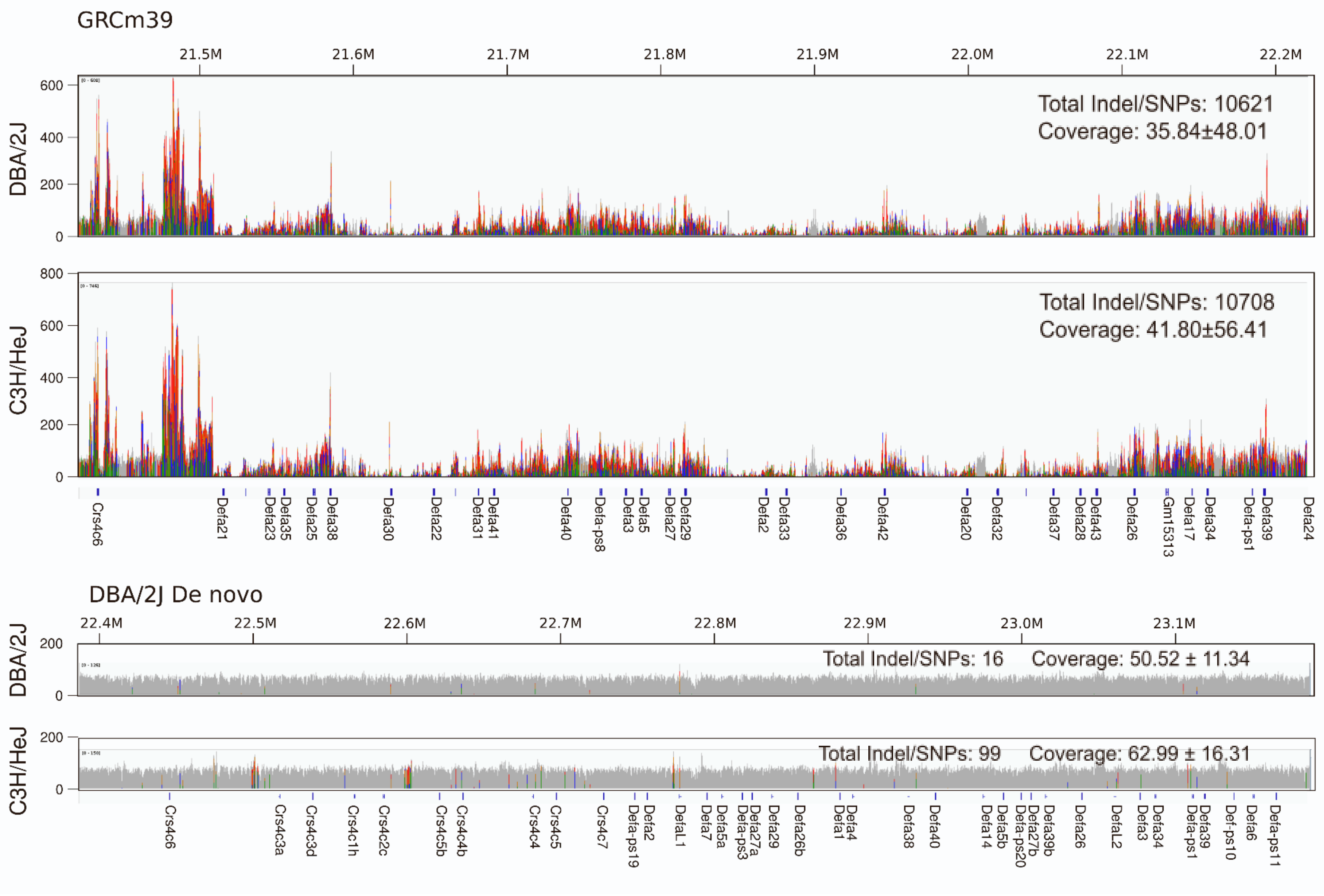
GRCm39
21.5M
21.6M
21.7M
21.8M
21.9M
22.0M
22.1M
22.2M
DBA/2J
600
400
200
0
Total Indel/SNPs: 10621
Coverage: 35.84±48.01
C3H/HeJ
800
600
400
200
0
Total Indel/SNPs: 10708
Coverage: 41.80±56.41
Crs4c6
Defa21
Defa23
Defa35
Defa25
Defa38
Defa30
Defa22
Defa31
Defa41
Defa40
Defa-ps8
Defa3
Defa5
Defa27
Defa29
Defa2
Defa33
Defa36
Defa42
Defa20
Defa32
Defa37
Defa28
Defa43
Defa26
Gm15313
Defa17
Defa34
Defa-ps1
Defa39
Defa24
DBA/2J De novo
22.4M
22.5M
22.6M
22.7M
22.8M
22.9M
23.0M
23.1M
DBA/2J
200
0
Total Indel/SNPs: 16
Coverage: 50.52 ± 11.34
C3H/HeJ
200
0
Total Indel/SNPs: 99
Coverage: 62.99 ± 16.31
Crs4c6
Crs4c3a
Crs4c3d
Crs4c1h
Crs4c2c
Crs4c5b
Crs4c4b
Crs4c4
Crs4c5
Crs4c7
Defa-ps19
Defa2
DefaL1
Defa7
Defa5a
Defa-ps3
Defa27a
Defa29
Defa26b
Defa1
Defa4
Defa38
Defa40
Defa14
Defa5b
Defa-ps20
Defa27b
Defa39b
Defa26
DefaL2
Defa3
Defa34
Defa-ps1
Defa39
Def-ps10
Defa6
Defa-ps11

Supplementary Figure 6: Whole genome Illumina raw reads from DBA/2J and C3H/HeJ realigned to α-defensin locus (~800Kb) highlighted in Figure 2c, visualized via IGV. (a) Alignment onto GRCm39 reference. There are large amounts of heterozygous SNPs, and many genome regions show very high (600+) or low coverage, which indicates gene loss or copy number variation. (b) The same reads are aligned to the DBA/2J *de novo* assembly. The figures showed a stable coverage and very few SNPs. This indicates that all α-defensin raw reads from both strains are properly mapped. Some additional heterozygous SNPs at 22.5M in strain C3H/HeJ are caused by a strain-specific LTR repeat element.

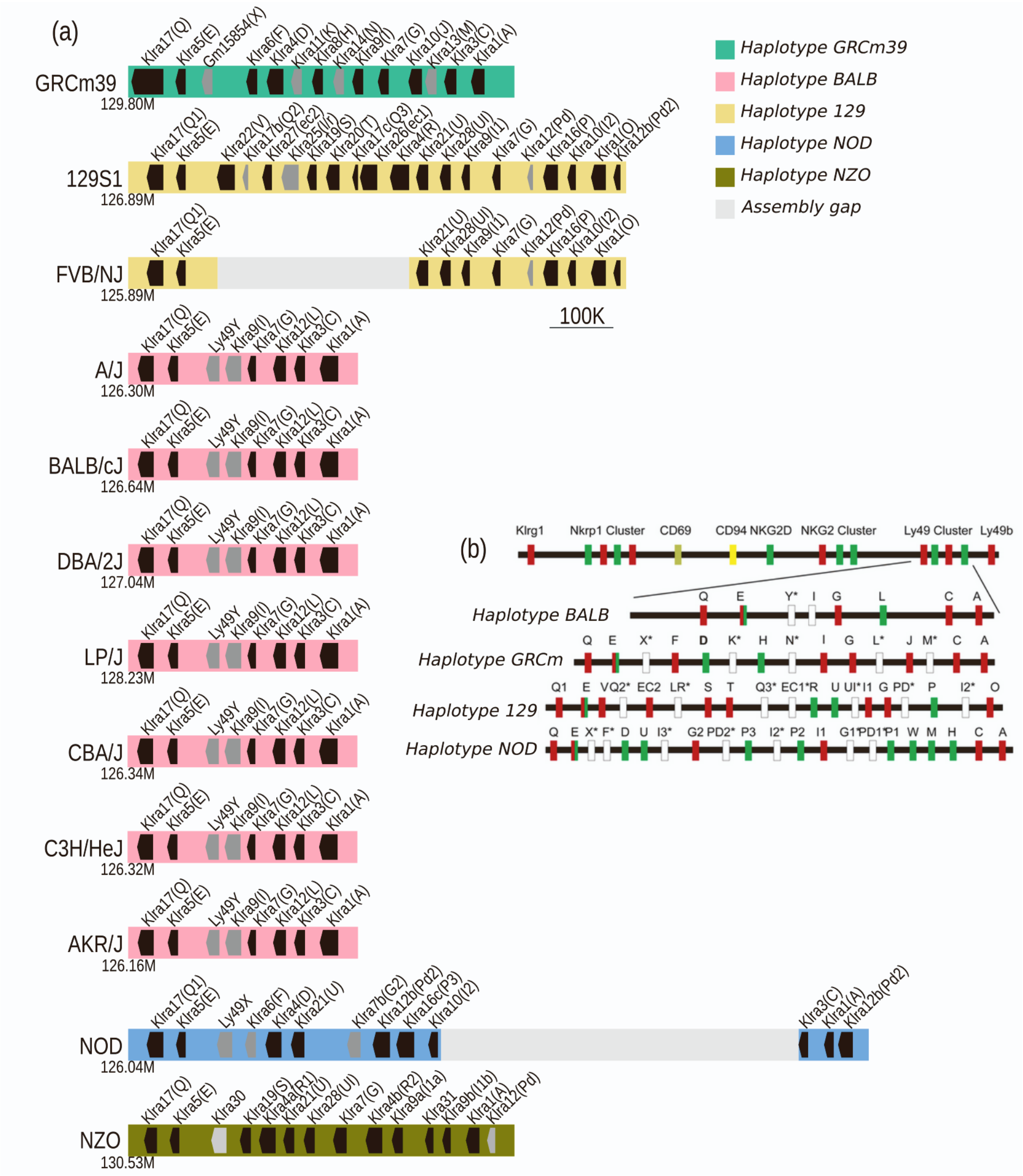

Supplementary Figure 7: Further to Figure 2 a/b/c, genome structure of Klra (Ly49) locus among 12 laboratory mouse strains. (a) Five haplotypes of Ly49 and the genes encoded. Black blocks indicate coding genes, and grey indicate pseudogenes. Arrows indicate the direction of the coding sequence. (b) Ly49 structure and annotation in the previous publication.

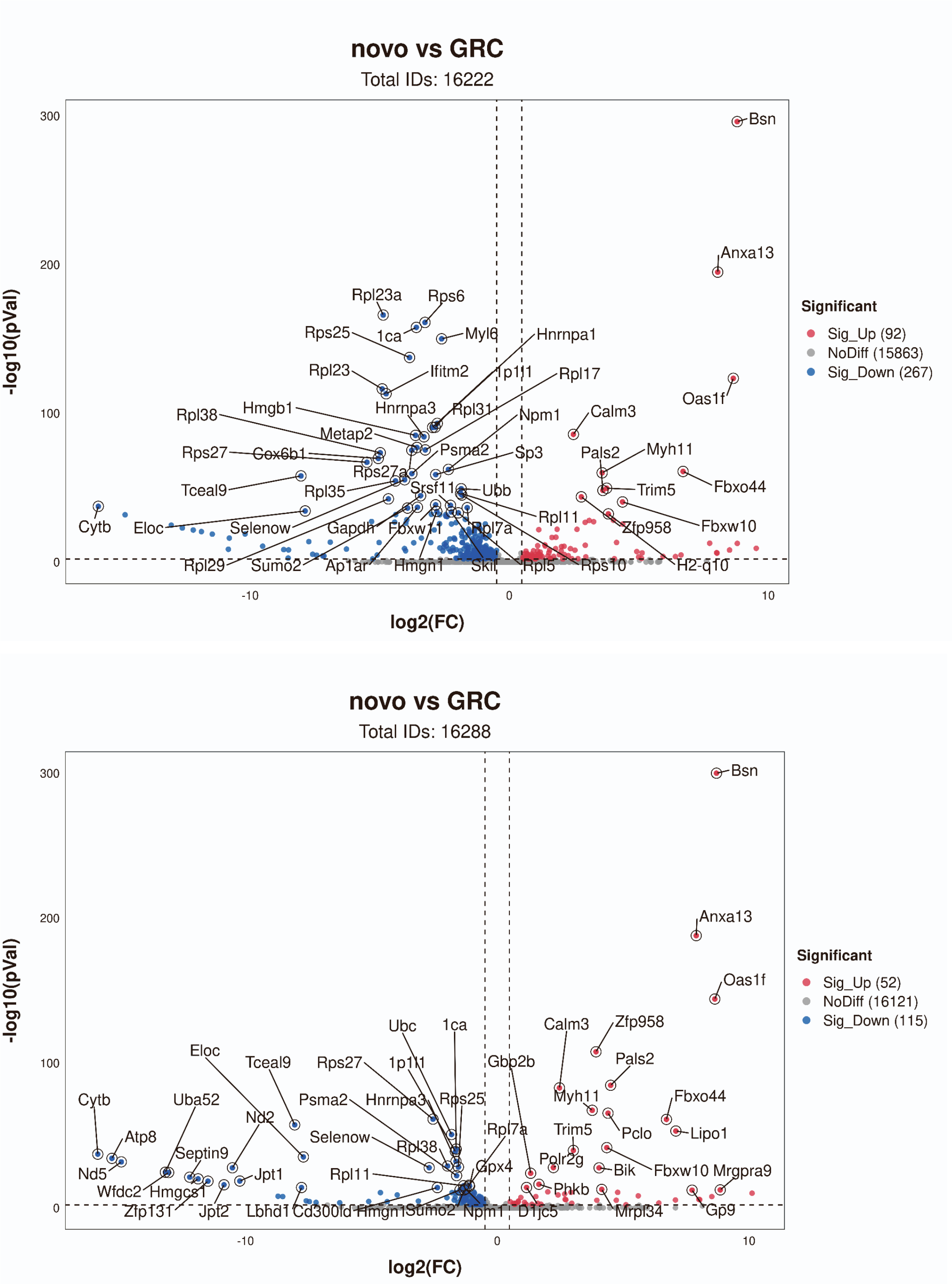

Supplementary Figure 8: Expanding Figure 4b, *Upper*, in 16222 annotated genes with unique paralogues between GRCm39 and PWK de novo, 2.1% of the genes show significant DE between different references at padj < 0.01 and log2(FC) > 0.5. However, most of the DE results are caused by multiple alignments between expressed genes and pseudogenes. *Lower*, when multiple alignments are permitted, this number is reduced to about 1%. Please note this analysis has excluded most gene family members and all novel genes in the non-reference strain.

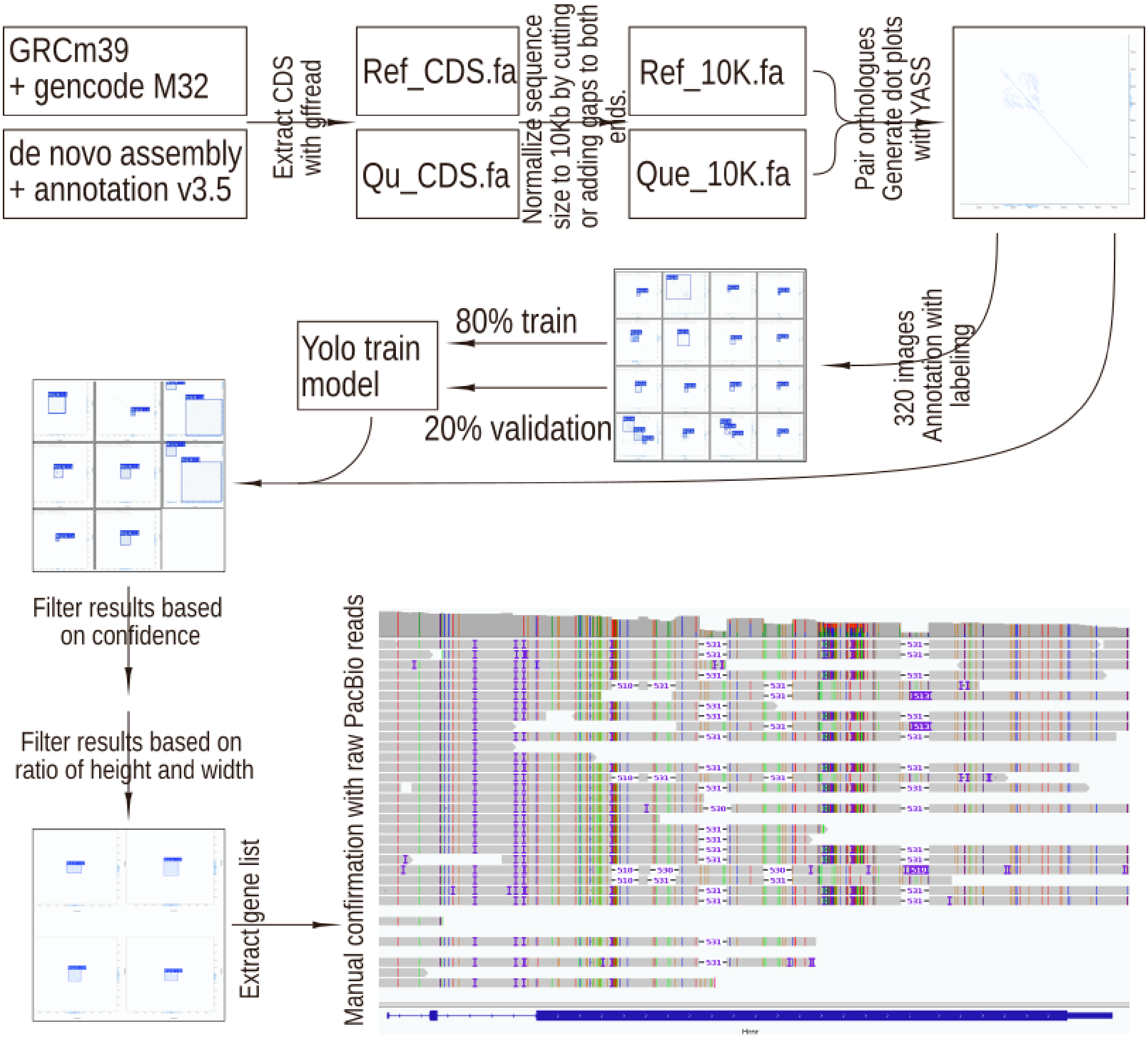

Supplementary Figure 9: A summary of image segmentation process used for the VNTR analysis (see methods).
